# Growth of *Corynebacterium glutamicum* on 5-oxo-L-proline (pyroglutamate) as a carbon and nitrogen source requires the PxpR-controlled *pxpTABC* genes

**DOI:** 10.1128/aem.02181-25

**Published:** 2026-05-29

**Authors:** Lea Sundermeyer, Christina Mack, Susana Matamouros, Michael Bott

**Affiliations:** 1 IBG-1: Biotechnology, Institute of Bio- and Geosciences, Forschungszentrum 685230 , Jülich, Germany; 2 Bioeconomy Science Center (BioSC), Forschungszentrum Jülich 685230 , Jülich, Germany; Washington University in St Louis, St. Louis, Missouri, USA

**Keywords:** *Corynebacterium glutamicum*, 5-oxoprolinase, transport, transcriptional regulation, isothermal titration calorimetry, biosensor

## Abstract

5-Oxo-L-proline (5-OP) is generated by spontaneous cyclization of L-glutamate and L-glutamine and is, therefore, expected to occur in amino acid-rich environments as well as in all cells. However, its utilization as a substrate by bacteria has rarely been examined. Here, we show that the actinobacterium *Corynebacterium glutamicum* grows efficiently in minimal medium with 5-OP as the sole carbon and nitrogen source. Under these conditions, the *pxpTABC* gene cluster was essential. *pxpT* encodes a secondary transporter of the APC superfamily that likely mediates 5-OP uptake, whereas *pxpABC* encode a recently identified ATP-dependent 5-oxoprolinase that converts 5-OP to L-glutamate for further metabolism. Both PxpT and PxpABC were required for growth on 5-OP. Upstream of and divergently transcribed from *pxpT* is *pxpR*, which encodes a GntR-type transcriptional regulator. Deletion of *pxpR* enhanced growth on 5-OP, suggesting that PxpR represses the expression of the *pxpTABC* operon. This conclusion was supported by reporter gene analyses and identification of the PxpR-binding site. In addition, isothermal titration calorimetry showed that purified PxpR binds 5-OP with a K_D_ of 726 ± 23 nM. In contrast, L-proline, L-glutamate, L-glutamine, and L-aspartate were not bound under the conditions tested, indicating that PxpR functions as a specific 5-OP sensor. These findings identify a dedicated uptake, catabolic, and regulatory system for 5-OP utilization in *C. glutamicum*.

**IMPORTANCE:**

Bacterial utilization of 5-oxoproline (5-OP) as a carbon and nitrogen source has received little attention. Because 5-OP forms spontaneously from L-glutamate and L-glutamine, it is likely ubiquitous in amino acid-rich environments as well as inside cells. In many bacteria, including *Corynebacterium glutamicum, Escherichia coli*, and *Bacillus subtilis*, intracellular L-glutamate concentrations can reach 100 mM or higher, favoring 5-OP formation. Here, we show that *C. glutamicum* uses 5-oxoprolinase (PxpABC) together with the transporter PxpT to grow on 5-OP as the sole carbon and nitrogen source. The widespread distribution of pxpABC genes further suggests that the capacity to exploit 5-OP may be common among bacteria and could contribute to growth and survival in diverse habitats.

## INTRODUCTION

5-Oxo-L-proline (5-OP), also termed pyroglutamate, is the cyclic lactam of glutamate. Its presence in all cells is inevitable due to the spontaneous cyclization of L-glutamate, L-glutamine, and γ-glutamyl phosphate (1). In the case of L-glutamine, approximately 10% is converted to 5-OP per day at 37°C with ammonia as the second product (2, 3). 5-OP is also formed enzymatically in the degradation of glutathione within the γ-glutamyl cycle (4) and by cleavage of N-terminal 5-OP residues of proteins by pyroglutamyl peptidases (5, 6). 5-OP is also present in amino acid-containing habitats, such as the rhizosphere (7) or in fermented products, such as fish sauce (8) or enzymatic hydrolysates of wheat gluten (9).

Earlier reports on 5-OP utilization and metabolism by bacteria were reviewed by van der Werf and Meister (10). A systematic study on the effects of 5-OP on different prokaryotic species revealed that growth of thermoacidophilic archaea and some bacteria was severely inhibited by the presence of 5-OP and that the extent of inhibition increased with increasing growth temperature and decreasing pH (11). For example, 15 mM 5-OP completely inhibited growth of *Sulfolobus solfataricus* at 78°C and pH 3 (12). In contrast, growth of some organisms living at neutral pH and moderate temperatures was not inhibited and sometimes was even stimulated by 5-OP (11). In halophilic and alkaliphilic methanotrophs, 5-OP can serve as an osmoprotectant (13). For *Salmonella enterica* serovar Typhimurium, it was reported that 5-OP cannot be used as a nutrient source for growth but has an influence on cellular aggregation (14).

5-OP formed during degradation of glutathione in eukaryotes is known to be converted to glutamate in an ATP-dependent reaction catalyzed by 5-oxoprolinase (15). An ATP-independent 5-oxoprolinase was identified in *Alcaligenes faecalis* (16). However, the equilibrium constant of the reaction, K_eq_ ([L-glutamate]/[5-OP]), was found to be ~0.035, showing that the formation of 5-OP from L-glutamate is highly favored. Interestingly, sequencing of the corresponding gene revealed that the ATP-independent 5-oxoprolinase contains a Sec signal peptide and, therefore, is presumably active in the periplasm (17). In a recent study, it was shown that of 984 analyzed bacterial and archaeal genomes, only 115 contain homologs of the eukaryotic-type 5-oxoprolinase and that only 10 had homologs of the enzyme of *A. faecalis* (18). The majority of the analyzed genomes were found to encode another type of ATP-dependent 5-oxoprolinase, showing no sequence similarity to the eukaryotic-type enzyme. It is composed of three subunits, and the corresponding genes were named *pxpA*, *pxpB,* and *pxpC* for prokaryotic 5-oxoprolinase (18). This enzyme was originally identified in *Pseudomonas* sp. (19). It is assumed that PxpB and PxpC are responsible for ATP-dependent phosphorylation of 5-OP, whereas PxpA catalyzes the decyclization to γ-glutamyl phosphate and subsequent dephosphorylation to L-glutamate (20, 21).

Studies with *Bacillus subtilis* revealed that the *pxpABC* genes are essential for utilization of 5-OP as the sole nitrogen source in minimal medium, but their absence also had a negative effect on growth, with ammonia as the nitrogen source (18). This suggests that the absence of 5-oxoprolinase also impairs the growth fitness of *B. subtilis* in the absence of exogenous 5-OP. It was shown that *B. subtilis* mutants lacking *pxpA*, *pxpB*, or *pxpC* accumulate 5-OP both within the cells and in the medium, whereas the wild-type does not (18). Therefore, the negative effect on growth observed for these mutants might be due to 5-OP accumulation. 5-OP can be used as the sole nitrogen source by *B. subtilis*, but not by mutants lacking the 5-oxoprolinase genes (18). Recently, the role of *pxp* genes was explored in *Clostridioides difficile* (22). In contrast to *B. subtilis*, the genes showed no effect on sporulation in *C. difficile* but were necessary for the growth-promoting effect of 5-OP observed for the wild-type of this anaerobic enteric pathogen. It was suggested that the *pxpAGBC* genes facilitate the use of 5-OP as a carbon source, nitrogen source, or both a carbon and nitrogen source (22).

In this study, we analyzed the fate of 5-OP in *Corynebacterium glutamicum*. This actinobacterial species is a model organism in microbial biotechnology since it is the most important industrial amino acid producer, with L-glutamate and L-lysine being produced at a scale of several million tons per year (23–26). Also, L-glutamine-producing strains of *C. glutamicum* have been developed (27). Although both L-glutamate and L-glutamine spontaneously cyclize to form 5-OP, the metabolism and use of this metabolite have never been studied in *C. glutamicum*. We show that *C. glutamicum* is able to grow with 5-OP as the sole carbon and nitrogen source and delineate the molecular basis. 5-OP, when present in the environment, is taken up by the secondary transporter PxpT. Cytoplasmic 5-OP is converted to L-glutamate by the 5-oxoprolinase PxpABC. The corresponding operon *pxpTABC* is regulated by a GntR-type transcriptional regulator, which we named PxpR and which specifically binds 5-OP with high affinity.

## RESULTS

### Growth of *C. glutamicum* with 5-OP as a sole carbon and nitrogen source

**Fig 1:**
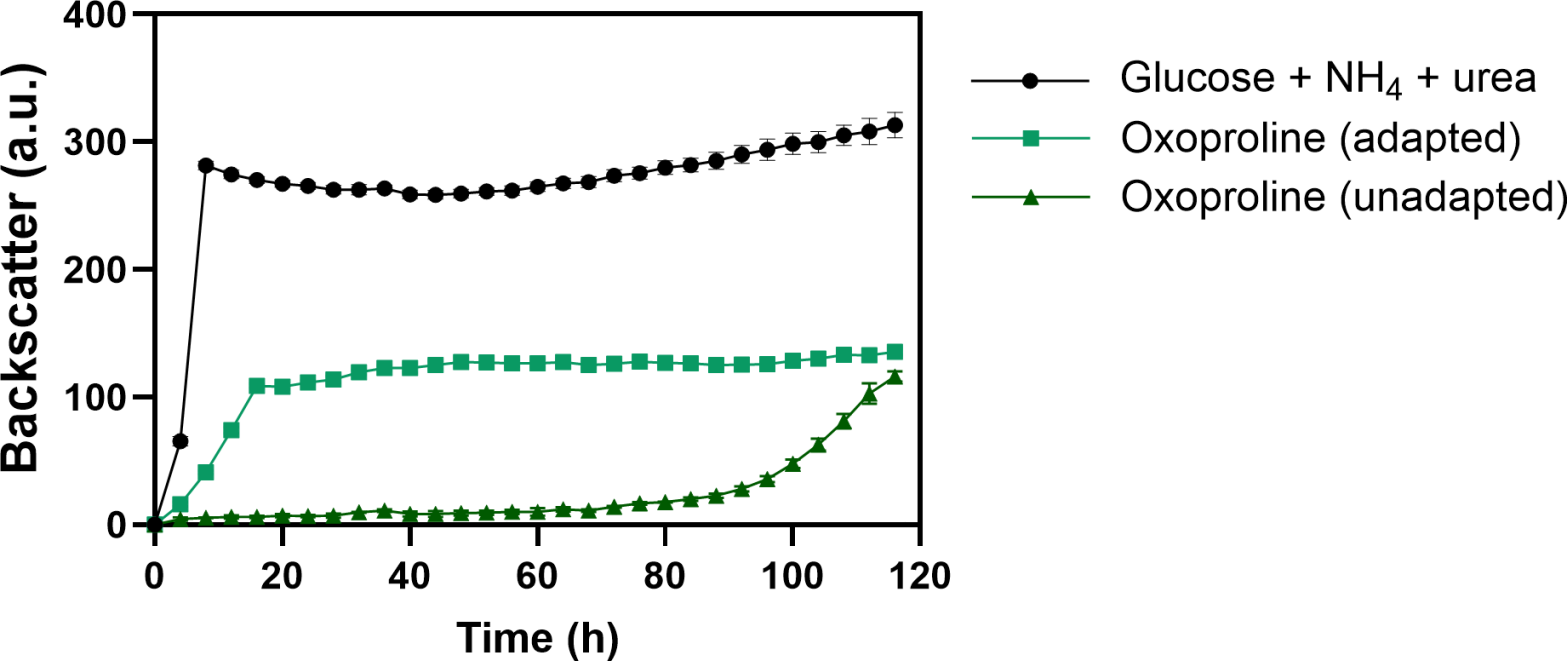
Growth of *C. glutamicum* WT on 5-OP as the sole carbon and nitrogen source. Cells were cultivated either in standard CGXII medium with 20 g/L glucose as the carbon source and 20 g/L ammonium sulfate plus 5 g/L urea as nitrogen sources or in modified CGXII medium lacking glucose, ammonium sulfate, and urea and containing 12.9 g/L 5-OP as the sole carbon and nitrogen source. The pre-cultures were grown either in glucose medium (unadapted) or in 5-OP medium (adapted). Cultivation was performed in a BioLector microcultivation system at 30°C and 1,200 rpm.

5-OP is an unavoidable metabolite in amino acid-containing habitats, and all cells are formed by spontaneous cyclization of L-glutamate, L-glutamine, or γ-glutamyl phosphate. Since the cytoplasmic concentration of L-glutamate in *C. glutamicum* cells has been reported to be in the range of 100–200 mM or even above (28–31), it can be expected that 5-OP is constantly formed. No studies have been performed so far on the fate of this metabolite, which prompted us to analyze how *C. glutamicum* deals with 5-OP. Initially, we tested if 5-OP can serve as a carbon and nitrogen source. As shown in Fig. 1, cultivation of the wild-type ATCC 13032 (WT) in modified CGXII minimal medium containing 12.9 g/L (100 mM) 5-OP as the sole carbon and nitrogen source enabled growth. As a control, standard CGXII medium with 20 g/L (NH_4_)_2_SO_4_ and 5 g/L urea as nitrogen sources and 20 g/L glucose (100 mM) as a carbon source was used. The lag phase for growth on 5-OP was long if the cells were pre-grown in glucose minimal medium but absent if cells were pre-cultured with 5-OP, suggesting that the genes required for 5-OP utilization are inducible (Fig. 1). In summary, *C. glutamicum* has the ability to utilize environmental 5-OP.

### Genes required for growth of *C. glutamicum* on 5-OP

**Fig 2:**
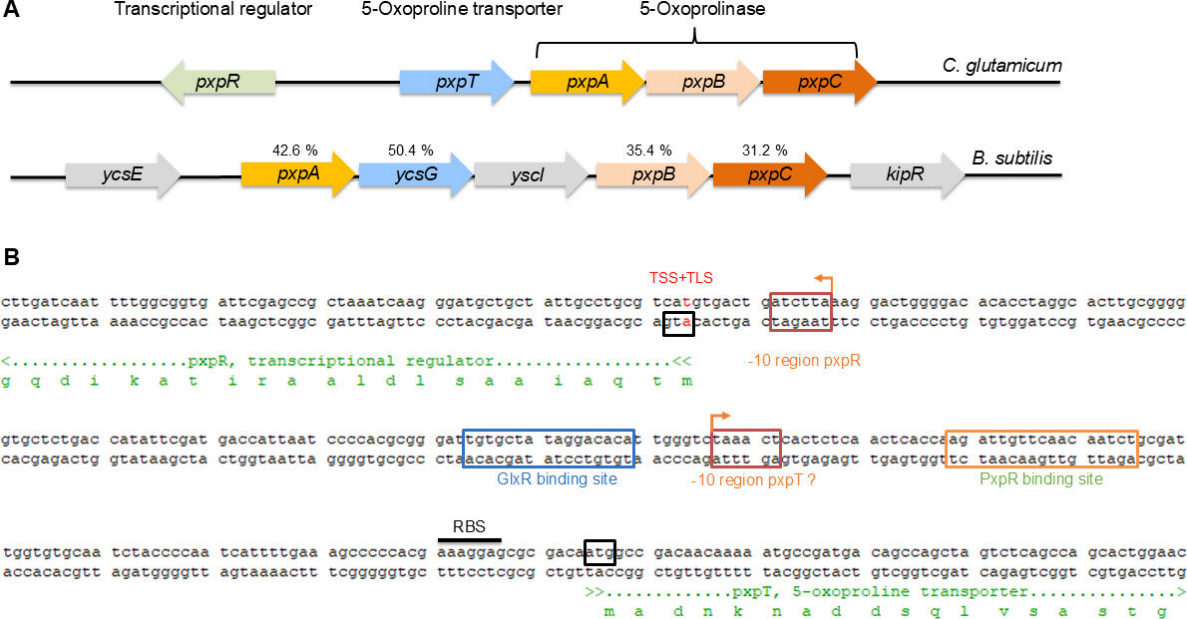
(**A**) Map of the *C. glutamicum* genome region with the 5-oxoprolinase genes *pxpABC* and, for comparison, the corresponding region of *B. subtilis*. The %-numbers indicate the protein sequence identity to the homologous *C. glutamicum* proteins. (**B**) DNA sequence showing the *pxpR-pxpT* intergenic region including the initial coding sequences. The locations of the PxpR-binding site, the GlxR-binding site, and of promoter elements are indicated. The −10 region of *pxpT* is predicted. The transcriptional start site (TSS) of *pxpR* was identified in a genome-wide study at the “a” nucleotide of the ATG start codon (translational start site, TLS) of *pxpR* (34), indicating that *pxpR* is a leaderless gene (35).

Inspection of the genome of *C. glutamicum* (32, 33) for genes encoding 5-oxoprolinase revealed a cluster of three genes encoding proteins showing 31%–43% amino acid sequence identity to the PxpA, PxpB, and PxpC proteins of *B. subtilis* (18). These genes were, therefore, named *pxpA* (cg1141)*, pxpB* (cg1140), and *pxpC* (cg1139). Upstream of *pxpA*, the gene *cg1142* is located in the same orientation (Fig. 2A) and encodes a protein with 50% sequence identity to YcsG of *B. subtilis*, which is present downstream of *pxpA* and was shown to be required for 5-OP utilization as a nitrogen source in *B. subtilis* (18). Based on the experiments reported below, cg1142 was named *pxpT* to indicate its function in 5-OP transport.

**Fig 3:**
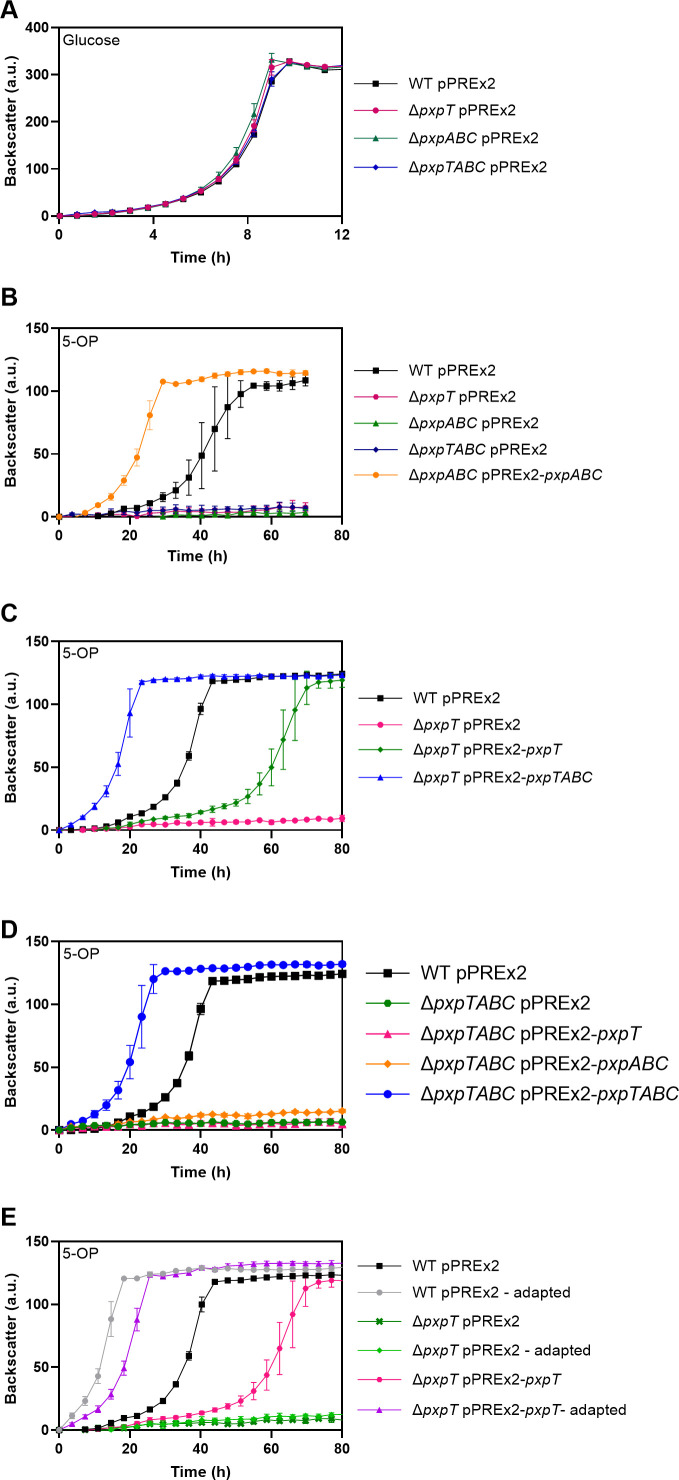
Dependency of growth with 5-OP on the *pxpTABC* genes. The indicated *C. glutamicum* strains were cultivated either in CGXII glucose medium as a control (**A**) or in modified CGXII medium containing 5-OP as the sole carbon and nitrogen source (**B–E**). All media contained 25 µg/mL kanamycin. “Adapted” indicates that the corresponding cultures were pre-grown in 5-OP medium, whereas all others were pre-grown in glucose medium. Cultivation was performed in a BioLector microcultivation system at 30°C and 1,200 rpm.

The relevance of *pxpABC* genes and of *pxpT* for 5-OP utilization was tested by constructing and analyzing the *C. glutamicum* deletion mutants Δ*pxpABC*, Δ*pxpTABC*, and Δ*pxpT*. All three mutants grew like the WT in standard CGXII minimal medium with glucose (20 g/L) (Fig. 3A) but were unable to grow with 5-OP (12.9 g/L) as the sole carbon and nitrogen source (Fig. 3B), suggesting that PxpABC and PxpT are essential for 5-OP utilization. For confirmation, the expression plasmids pPREx2-*pxpABC*, pPREx2-*pxpTABC*, and pPREx2-*pxpT* were constructed and used for transformation of the mutant strains. As shown in Fig. 3B, plasmid-based expression of *pxpABC* in the mutant strain Δ*pxpABC* not only restored growth on 5-OP but even improved growth (reduced the lag phase and increased the growth rate) compared to the WT. Similarly, plasmid-based expression of either *pxpT* alone or of *pxpTABC* recovered the growth of the Δ*pxpT* mutant on 5-OP; however, the expression of *pxpTABC* enabled markedly better growth than that of *pxpT* alone (Fig. 3C). Complementation of the Δ*pxpTABC* mutant with plasmid-encoded *pxpTABC* recovered growth on 5-OP, whereas complementation with *pxpABC* alone or *pxpT* alone did not (Fig. 3D). These results confirm that both *pxpT* and *pxpABC* are required for growth of the Δ*pxpTABC* mutant on 5-OP. We also tested whether pre-cultivation on 5-OP influences the growth of the Δ*pxpT* strain with pPREx2-*pxpT*. As shown in Fig. 3E, precultivation on 5-OP strongly reduced the lag phase and increased the growth rate, suggesting that in this strain, the *pxpABC* genes remain inducible by 5-OP.

### Regulation of the *pxpTABC* genes by PxpR

**Fig 4:**
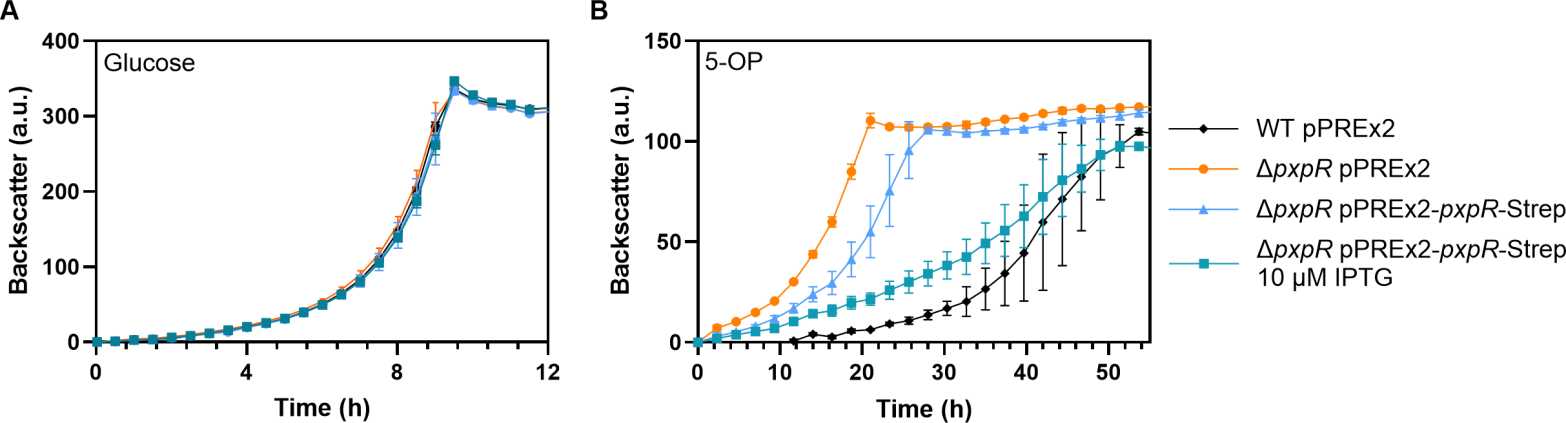
Influence of the transcriptional regulator PxpR on growth with 5-OP. The indicated *C. glutamicum* strains were cultivated either in CGXII glucose medium as control (**A**) or in modified CGXII medium containing 5-OP as the sole carbon and nitrogen source (**B**), all containing 25 µg/mL kanamycin. Where indicated, the medium contained 10 µM IPTG for enhancing the expression of *pxpR*-Strep.

Upstream and divergently oriented to *pxpT*, a gene (cg1143) encoding a transcriptional regulator of the GntR family (36) is located and was designated *pxpR* (Fig. 2). PxpR consists of an N-terminal helix-turn-helix (HTH) domain (residues 11–69) and a C-terminal ligand-binding domain (residues 79–205). Due to its genomic localization, an involvement of PxpR in transcriptional regulation of the *pxpTABC* genes appeared likely and was tested by the construction and analysis of a Δ*pxpR* deletion mutant. As shown in Fig. 4, the absence of PxpR had no effect on growth on glucose, but it strongly improved growth on 5-OP by shortening the lag phase and increasing the growth rate. Complementation of the Δ*pxpR* deletion mutant with plasmid pPREx2-*pxpR*-Strep inhibited growth on 5-OP, and this inhibition became stronger when *pxpR* expression was increased by IPTG addition. These results suggest the function of PxpR as a repressor of *pxpTABC,* whose absence leads to constitutive expression of the genes.

To confirm the repression of *pxpTABC* by PxpR, the *pxpT* promoter region (212 bp upstream of the *pxpT* start codon) was fused to the *venus* reporter gene, resulting in plasmid pJC1-P*_pxpT_*-Venus. The plasmid was introduced into the WT and the Δ*pxpR* mutant, and the two strains were cultivated in standard CGXII glucose medium. Both strains showed the same growth behavior, but the specific Venus fluorescence (ratio fluorescence/backscatter) was 3-fold higher for the Δ*pxpR* mutant (0.177 ± 0.005) than for the wild-type (0.063 ± 0.018), supporting the role of PxpR as a repressor for the expression of the *pxpT* promoter.

The RegPrecise database (37), which predicts transcriptional regulons based on comparative genomics (37), proposes a 17-bp inverted repeat sequence as a DNA-binding site for PxpR (Fig. S1). This motif is centered 65 bp upstream of the start codon of *pxpT* (Fig. 2B). A putative −10 region (TAACCT) for *pxpT* was identified 95.5 bp upstream of the *pxpT* start codon, supporting a repressor function of PxpR. The transcriptional start site of *pxpR* was identified in a genome-wide study at the ATG start codon of *pxpR* (34), indicating that *pxpR* is a leaderless gene (35). Due to the 143-bp distance of the predicted PxpR-binding site to the transcriptional start site of *pxpR*, autoregulation of *pxpR* expression appears unlikely.

**Fig 5:**
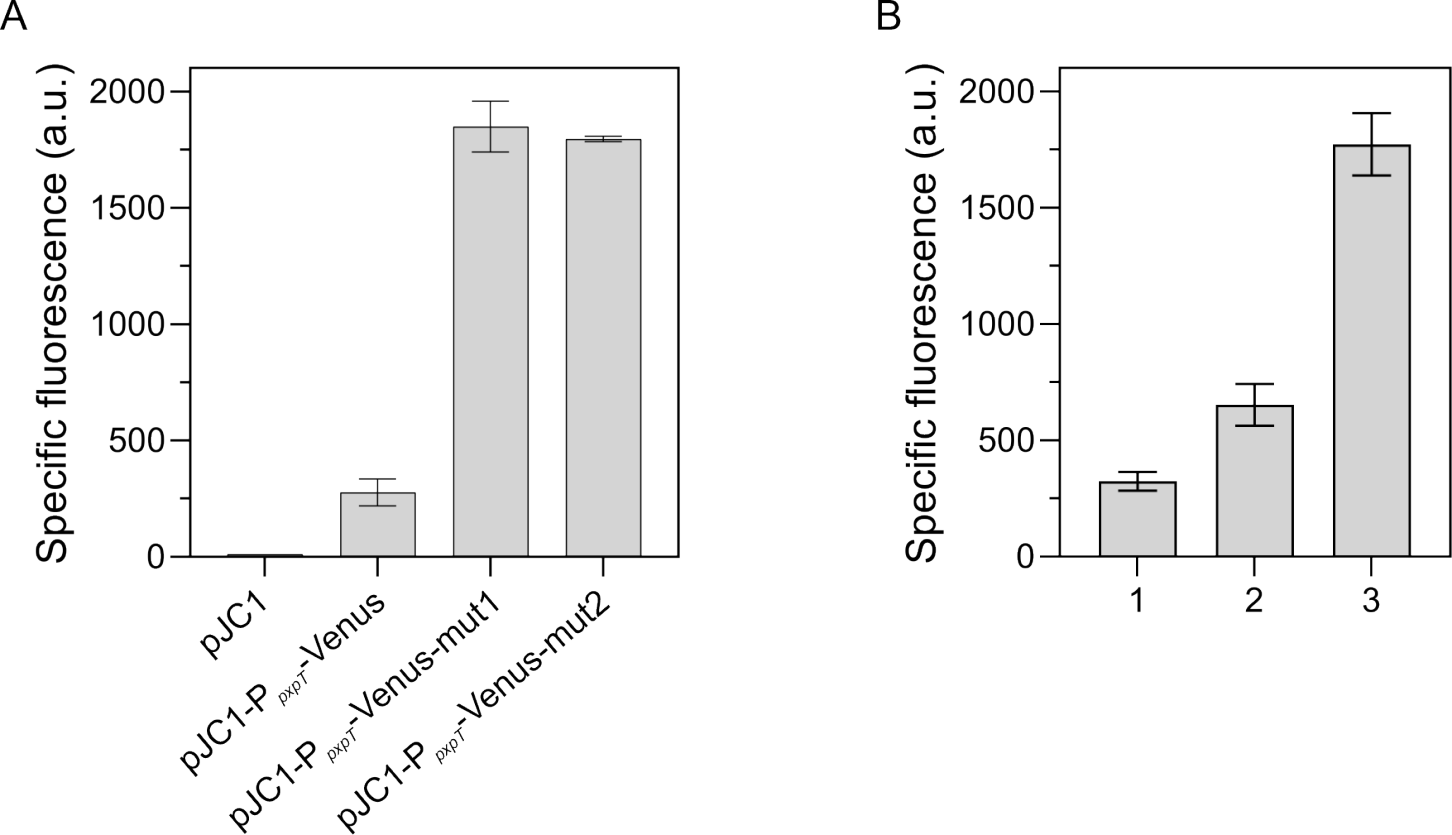
(**A**) Influence of the mutated (pJC1-P*_pxpT_*-Venus-mut1) or deleted (pJC1-P*_pxpT_*-Venus-mut2) proposed DNA-binding motif of PxpR on the expression of the reporter gene *venus* under control of the *pxpT* promoter in *C. glutamicum* WT. The strains were grown in CGXII glucose medium, and the specific fluorescence (ratio Venus fluorescence (Ex_515_ Em_528_)/OD_600_) in stationary-phase cells is shown. (**B**) Influence of 5-OP on the expression of the reporter gene *venus* under control of the *pxpT* promoter in *C. glutamicum* WT. The specific fluorescence of cultures grown until the stationary phase in standard CGXII glucose medium without 5-OP (1), in modified CGXII medium containing glucose and 5-OP as the sole nitrogen source (2), and in modified CGXII medium with 5-OP as the sole carbon and nitrogen source (3) is shown. For panels **A** and **B**, mean values of three biological replicates and standard deviations are shown.

To test the relevance of the proposed DNA sequence motif for expression of the *pxpTABC* genes, two mutated variants of plasmid pJC1-PpxpT-Venus were constructed, one in which the native sequence motif 5′-agattgttcaacaatct-3′ was changed to 5′-aggccacccggtggcct-3′ (pJC1-P_pxpT_-Venus-mut1) and one in which the native sequence motif 5′-agattgttcaacaatct-3′ was deleted (pJC1-P_pxpT_-Venus-mut2). When the WT transformed with the original or the mutated reporter plasmids was cultivated in glucose minimal medium, the reporter with the native promoter sequence showed a specific fluorescence of ~277 units, probably triggered by internally formed 5-OP. The reporter strains with the mutated or deleted DNA sequence motif had a 6.5-fold higher specific fluorescence (~1,800 units), supporting the assumption that this motif is the binding site for the PxpR repressor (Fig. 5A).

In a further experiment, we tested the influence of 5-OP on the expression of the *pxpT* promoter using pJC1-P_pxpT_-Venus. Three cultivation conditions were compared, i.e., regular CGXII medium with glucose, ammonium sulfate, and urea (condition 1); modified CGXII medium with glucose and 5-OP as the sole nitrogen source (condition 2); and modified CGXII medium with 5-OP as the sole carbon and nitrogen source (condition 3). As shown in Fig. 5B, when compared to the cultivation condition in standard CGXII medium without 5-OP addition (~320 units), the reporter gene expression increased ~2-fold (~650 units) when 5-OP served as the sole nitrogen source and about 5.5-fold (~1770 units) when 5-OP served as the sole carbon and nitrogen source. These results indicate that the expression of the *pxpT* promoter is induced in the presence of 5-OP. The observation that the expression level of the reporter was lower in the presence of glucose and 5-OP when compared to the condition where 5-OP served as both the sole carbon and nitrogen source and glucose was absent suggests that the *pxpTABC* genes could be controlled by additional transcription factors besides PxpR.

### PxpR functions as a biosensor for 5-OP

**Fig 6:**
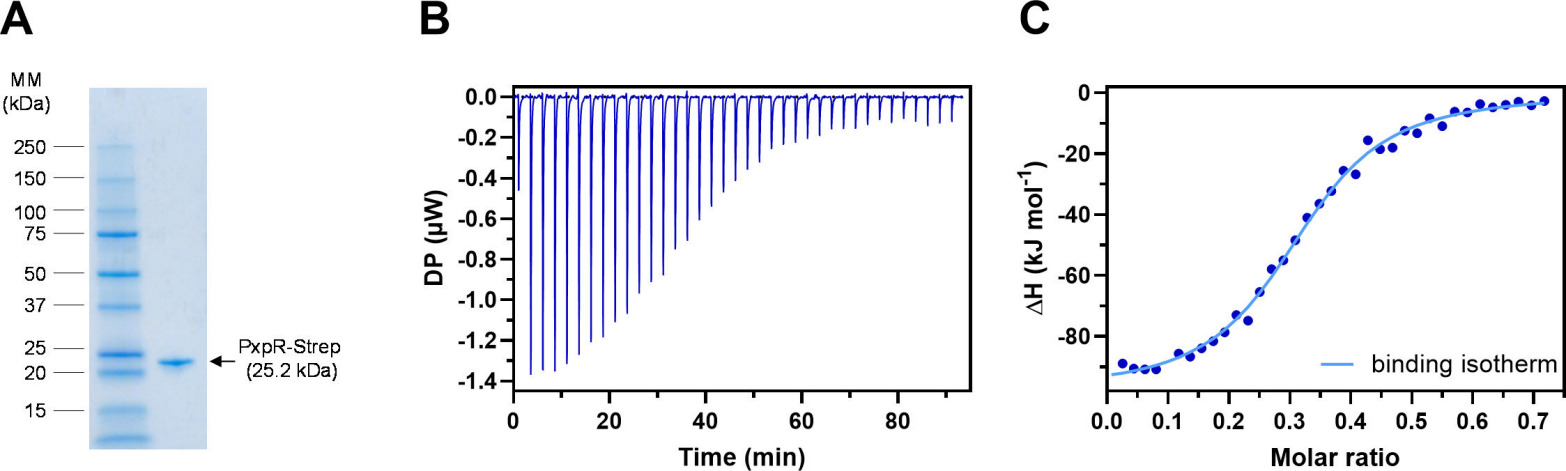
Purification of PxpR and ligand-binding studies by isothermal titration calorimetry. (**A**) SDS-PAGE of PxpR-Strep purified by Strep-tag HP affinity and size exclusion chromatography. (**B**) Raw data for a representative ITC experiment with 40 µM PxpR-Strep and titration with 150 µM 5-OP. (**C**) Binding isotherm calculated from the raw data shown in panel **B**. DP, differential power.

Based on the results described above, it appeared likely that PxpR senses the 5-OP concentration in the cytoplasm and that binding of 5-OP to PxpR triggers a conformational change that leads to derepression of the *pxpTABC* genes. To test for 5-OP binding to PxpR, the protein modified by a carboxyterminal Strep-tag was overproduced in *E. coli* BL21(DE3) and purified by StrepTactin affinity chromatography, followed by size-exclusion chromatography (Fig. 6). The purified protein was used for binding studies using isothermal titration calorimetry (ITC), which measures binding interactions by detecting the heat absorbed or released during a binding event (38, 39). Deconvolution of the binding isotherm gives the number of binding sites (n), the enthalpy of binding (ΔH), and the equilibrium dissociation constant (K_D_). From these parameters, the Gibbs free energy (ΔG) and the entropy of binding (ΔS) can be derived. The ITC experiments (Fig. 6 and Table S1) showed that PxpR binds 5-OP with a K_D_ of 726 ± 23 nM (mean value of five determinations). Binding of 5-OP is an exothermic event with a ΔH of −98.7 ± 1.0 kJ mol^−1^ and a loss of entropy (-TΔS = 63.6 ± 1.1 kJ mol^−1^). The binding stoichiometry was 0.324 ± 0.004 if each PxpR monomer binds one molecule of 5-OP, suggesting that a fraction of the protein was in a binding-incompetent state. Importantly, L-glutamate, L-aspartate, L-glutamine, and L-proline did not bind to PxpR under the same experimental conditions (data not shown), suggesting that PxpR serves as a specific 5-OP biosensor.

## DISCUSSION

**Fig 7:**
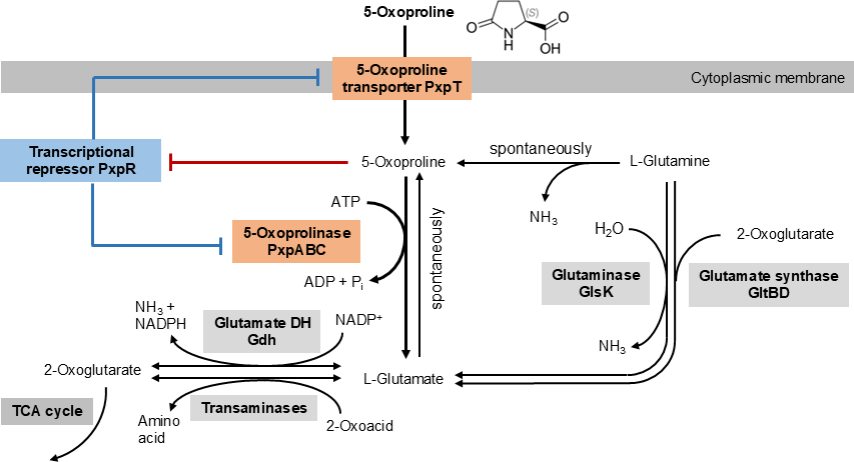
Overview on the formation and degradation of 5-OP in *C. glutamicum* and transcriptional regulation by PxpR.

In this study, we analyzed the utilization of 5-OP by *C. glutamicum*. No studies have been performed to date on the fate of this metabolite in *C. glutamicum*; however, its presence in *C. glutamicum* cells was previously reported in a metabolomics study by GC-MS (40). We showed that *C. glutamicum* can grow with 5-OP as the sole carbon and nitrogen source and identified the genes required for this metabolic pathway and their transcriptional regulation. The assumed pathways for 5-OP formation and utilization are summarized in Fig. 7. Due to the widespread occurrence of the *pxpABC* genes (18), it can be assumed that the capability to utilize 5-OP as a carbon and nitrogen source is widespread in bacteria.

The *pxpT* gene encodes a secondary transporter of 436 amino acid residues with 11 predicted transmembrane helices. BLAST searches in the Transporter Classification Database (41) showed that the proteins with the highest sequence identity, such as YcsG of *B. subtilis*, belong to the NRAMP family of divalent metal ion transporters (TCDB 2.A.55). This family is part of the large amino acid-polyamine-organocation (APC) superfamily of secondary transporters, which also includes several families involved in amino acid transport (42). Although we cannot exclude a function of PxpT in the transport of divalent metal ions, our data suggest that PxpT is required for uptake of 5-OP by *C. glutamicum*. Deletion of *pxpT* prevented growth on 5-OP, which could be reversed by transformation of the Δ*pxpT* mutant with plasmid-encoded *pxpT*. The homologous YcsG protein of *B. subtilis* is encoded downstream of the *pxpA* gene, and deletion of *ycsG* prevented the use of 5-OP as a nitrogen source (18), supporting the function of YcsG in 5-OP uptake. A homolog of PxpT is also encoded in the recently analyzed *pxpAGBC* operon of *C. difficile* (PxpG, 46% amino acid sequence identity to PxpT), but its function has not been studied yet (22). The observation that lack of PxpT prevents 5-OP utilization suggests that the transporters involved in L-proline uptake in *C. glutamicum*, PutP (43), ProP, and EctP (44), cannot compensate for the absence of PxpT under the conditions used in our experiments. A different type of 5-OP uptake system has been described for *Bordetella pertussis*, which belongs to the tripartite ATP-independent periplasmic (TRAP) transport systems (45). The crystal structures of two extracytoplasmic solute receptors DctP6 and DctP7 were solved in complex with 5-OP. The K_D_ of DctP7 for 5-OP was found to be 0.3 µM. Although 5-OP utilization in *B. pertussis* could not be demonstrated, the fact that the genes BP1887 and BP1891 for DctP6 and DctP7, respectively, are located in the vicinity of a gene (locus tag BP1985) encoding a eukaryotic-type 5-oxoprolinase supports the existence of TRAP-type 5-OP uptake systems in some bacteria.

Similar to the *pxpT* gene, the *pxpABC* genes, encoding 5-oxoprolinase, were essential for growth of *C. glutamicum* on 5-OP as the sole carbon and nitrogen source. Interestingly, growth of mutants lacking *pxpABC* or *pxpTABC* on glucose was not impaired (Fig. 3A), although 5-OP can presumably no longer be converted to glutamate by 5-oxoprolinase and, therefore, might accumulate within the cells. As L-proline is known to serve as a compatible solute in *C. glutamicum* that can accumulate to very high concentrations (46), 5-OP may also be tolerated at high concentrations. As mentioned before, 5-OP serves as a compatible solute in halophilic and alkaliphilic methanotrophic bacteria, which contain high concentrations under hyperosmotic stress (13). In *Methylomicrobium alcaliphilum* cells grown at an NaCl concentration of 1 M, the concentration of 5-OP reached 0.4 M (47). Therefore, it seems possible that other bacteria like *C. glutamicum* can also tolerate high 5-OP concentrations.

To our knowledge, regulation of 5-OP metabolism in bacteria has not been studied before. We demonstrated that the *pxpTABC* genes of *C. glutamicum* are repressed by the GntR-type transcriptional regulator PxpR. Deletion of *pxpR* had no influence on growth with glucose but strongly improved growth on 5-OP by reducing the lag phase and increasing the growth rate. This phenotype of the Δ*pxpR* mutant could be reversed by plasmid-encoded expression of *pxpR,* whereby stronger expression led to stronger growth inhibition (Fig. 4). The repression of the *pxpTABC* genes by PxpR was supported by a plasmid-encoded reporter gene fusion of the *pxpT* promoter with *venus*, which revealed a threefold higher expression level in the Δ*pxpR* mutant than in the WT after growth on glucose. The expression observed in the WT is presumably triggered by endogenously formed 5-OP, leading to basal levels of PxpT and 5-oxoprolinase. When 5-OP was provided in the medium as the sole carbon and nitrogen source, the expression of the reporter gene was increased 5.5-fold, suggesting that uptake of 5-OP via PxpT leads to increased cytoplasmic 5-OP levels and stronger derepression of the *pxpT* promoter from PxpR. Interestingly, when glucose and 5-OP as the sole nitrogen source were provided in the medium, the basal level of the reporter gene was increased only twofold, indicating that glucose had a negative influence on *pxpTABC* expression. Such a negative influence might be caused by the cAMP-responsive global regulator GlxR (48–50). A putative DNA-binding site for GlxR (5′-TGTGCTATAGGACACA-3′) was identified in the intergenic region between *pxpR* and *pxpT* close to the assumed −10 region of *pxpT* (49, 51), suggesting a negative influence on *pxpTABC* expression. As the cAMP level was reported to be high in glucose-grown cells (48, 52), a repressor function of GlxR for *pxpTABC* appears likely. Although 5-OP can serve as the sole nitrogen source, the *pxpTABC* genes were not found to be part of the nitrogen starvation stimulon, which includes the regulon of the master regulator of the nitrogen starvation response, AmtR (53–56).

The function as a repressor of *pxpTABC* suggested that PxpR senses 5-OP and relieves repression of *pxpTABC* depending on the cytoplasmic concentration of 5-OP. In agreement with this suggestion, we could show by ITC that purified PxpR binds 5-OP with a K_D_ of 726 nM. This suggests that 5-OP concentrations in the µM range might be sufficient for derepression of *pxpTABC* genes. Since we could not detect the binding of L-glutamate, L-aspartate, L-glutamine, and L-proline under the tested conditions, PxpR is presumably a specific 5-OP sensor. This offers the opportunity to develop various types of 5-OP biosensors enabling cytoplasmic 5-OP detection, such as FRET-based biosensors (57).

In summary, our results suggest that the *pxpTABC* genes fulfill a dual role in *C. glutamicum*. On the one hand, they enable the cells to convert 5-OP formed spontaneously within the cell to L-glutamate and thus allow its re-utilization, and, on the other hand, the *pxpTABC* genes also enable the uptake of environmental 5-OP, which is likely present in amino acid-rich environments, and its utilization as a carbon and nitrogen source for growth and survival.

## MATERIALS AND METHODS

### Bacterial strains, media, and culture conditions

All bacterial strains and plasmids used in this work are listed in Table 1 and all oligonucleotides in Table 2. *Escherichia coli* cells were cultivated at 37 °C in lysogeny broth (LB) (58) or terrific broth (TB) (12 g/L tryptone, 24 g/L yeast extract, 4 mL glycerol, 12.54 g/L K_2_HPO_4_, and 2.31 g/L KH_2_PO_4_; pH 7.0) or on LB agar plates (Carl Roth, Karlsruhe, Germany). *C. glutamicum* strains were cultivated at 30°C in brain heart infusion medium (BHI; Difco Laboratories, Detroit, USA) or in CGXII medium with 20 g/L glucose (59) containing 30 mg/L 3,4-dihydroxybenzoate as an iron chelator or in modified CGXII medium lacking glucose, ammonium sulfate, and urea and containing 12.9 g/L 5-OP as the nitrogen and carbon source. Solid media were prepared by adding 15 g/L agar to these media. To maintain plasmid stability, kanamycin was added at concentrations of 25 µg/mL (*C. glutamicum*) or 50 µg/mL (*E. coli*).

**TABLE 1:** Bacterial strains and plasmids used in this study

Strain or plasmid	Description	Reference or source
*C. glutamicum* strains		
ATCC 13032	Biotin-auxotrophic wild-type strain	DSMZ
Δ*pxp*ABC	Wild-type derivative with in-frame deletion of *pxp*ABC (cg1141-cg1140-cg1139)	This work
Δ*pxp*TABC	Wild-type derivative with in-frame deletion of *pxp*TABC (cg1142-cg1141-cg1140-cg1139)	This work
Δ*pxp*T	Wild-type derivative with in-frame deletion of *pxpT* (cg1142)	This work
Δ*pxpR*	Wild-type derivative with in-frame deletion of *pxpR* (cg1143)	This work
*E. coli* strains		
DH5α	F- *supE44* Δ*lacU*169 (Φ80*lacZ*Δ*M15*) *hsdR17 recA1 endA1 gyrA96 thi-1 relA1*	(60)
BL21(DE3)	F- *ompT hsdSB(rB-mB-) gal dcm* (λcIts857 *ind1* Sam7 *nin5 lacUV5*-T7 Gen 1	(61)
Plasmids		
pK19mobsacB	Kan^R^; suicide vector for allelic exchange in *C. glutamicum*; *oriV*_*E. coli*_ *oriT sacB*	(62)
pK19mobsacB-∆pxpABC	Kan^R^; pK19mobsacB derivative containing a PCR product covering the upstream region of *pxpA* and the downstream region of *pxpC*	This work
pK19mobsacB-∆pxpTABC	Kan^R^; pK19mobsacB derivative containing a PCR product covering the upstream region of *pxpT* and the downstream region of *pxpC*	This work
pK19mobsacB-∆pxpT	Kan^R^; pK19mobsacB derivative containing a PCR product covering the upstream and downstream regions of *pxpT*	This work
pK19mobsacB-∆pxpR	Kan^R^; pK19mobsacB derivative containing a PCR product covering the upstream and downstream regions of *pxpR*	This work
pPREx2	Kan^R^; pPBEx2 derivative (P*_tac_*, *lacI*^q^, and *ori*_*Cg*_ from pBL1; *ori*_*Ec*_ ColE1 from pUC18), with a consensus RBS (AAGGAG) for *C. glutamicum*	(63)
pPREx2-pxpABC	Kan^R^; pPREx2 derivative carrying the *pxpABC* genes	This work
pPREx2-pxpTABC	Kan^R^; pPREx2 derivative carrying the *pxpTABC* genes	This work
pPREx2-pxpT	Kan^R^; pPREx2 derivative carrying the *pxpT* gene	This work
pPREx2-pxpR-Strep	Kan^R^; pPREx2 derivative carrying the *pxpR* gene fused to a Strep tag-II encoding sequence at the 3′-end	This work
pPREx6	Kan^R^; pPREx2 derivative (P_T7_, *lacI*^*q*^, and *ori*_*Cg*_ from pBL1; *ori*_*E*_*_c._* ColE1 from pUC18), with a consensus RBS (AAGGAG) for *C. glutamicum*	(64)
pPREx6-pxpR-Strep	pPREx6 derivative carrying the *pxpR* gene fused to a Strep tag-II encoding sequence at the 3′-end under the control of the T7 promoter	This work
pJC1	Kan^R^; *E. coli*-*C. glutamicum* shuttle vector	(65)
pJC1-P*pxpT*-Venus	Kan^R^; pJC1 derivative containing the Venus reporter gene under the control of the *pxpT* promoter (transcriptional fusion)	This work
pJC1- P_pxpT_-Venus-mut1	Kan^R^; pJC1-P_pxpT_-Venus variant in which the proposed PxpR-binding site 5′-agattgttcaacaatct-3′ was changed to 5′-aggccacccggtggcct-3′	This work
pJC1- P_pxpT_-Venus-mut2	Kan^R^; pJC1-P_pxpT_-Venus variant in which the proposed PxpR-binding site 5′-agattgttcaacaatct-3′ was deleted	This work

**TABLE 2:** Oligonucleotides used in this study

Name	Sequence
Construction of pK19mobsacB-∆*pxpABC*	
D1_cg1139-1141	TGCAGGTCGACTCTAGAGTCATGCGCGTGGTGCTCTTC
D2_cg1139-1141	GACTTTATCGCCCGGCAATTCGCCGAGGTCGCTGTTG
D3_cg1139-1141	AACAGCGACCTCGGCGAATTGCCGGGCGATAAAGTCAG
D4_cg1139-1141	GTAAAACGACGGCCAGTGTCAGGTGAGGGACATCTAC
Construction of pK19mobsacB-∆*pxpTABC*	
D1_cg1139-1142	TGCAGGTCGACTCTAGAGTAGGCCTCTCGCATTACCC
D2_cg1139-1142	GACTTTATCGCCCGGCAATGTCGCGCTCCTTTCGTG
D3_cg1139-1142	CACGAAAGGAGCGCGACATTGCCGGGCGATAAAGTCAG
D4_cg1139-1142	GTAAAACGACGGCCAGTGTCAGGTGAGGGACATCTAC
Construction of pK19mobsacB-∆*pxpT*	
D1_cg1142	TGCAGGTCGACTCTAGAGTAGGCCTCTCGCATTACCC
D2_cg1139-1142	AACCAGGCCGATGACTCCTGTCGCGCTCCTTTCGTG
D3_cg1142	CACGAAAGGAGCGCGACAGGAGTCATCGGCCTGGTTATC
D4_cg1142	GTAAAACGACGGCCAGTGAGCCAGGGCCATGAGGGAG
Construction of pK19mobsacB-∆*pxpR*	
D1_cg1143	TTGTTCCGCCTCTTCGAACATGTGACTGATCTTAAAGGAC
D2_cg1143	TGCAGGTCGACTCTAGAGTAACCGTGTTGCCGAGTTC
D3_cg1143	TTTAAGATCAGTCACATGTTCGAAGAGGCGGAACAATAC
D4_cg1143	GTAAAACGACGGCCAGTGAGCTGCATTCATCGCGAG
Confirmation of gene deletion by colony PCR	
Dcg1139-1142 rv	TGGCAGAACCGACAGTTTG
Dcg1139-1142 fw	GTAAAACGACGGCCAGTGTCAGGTGAGGGACATCTAC
Dcg1143 fw	TCCAGTGCTGGCTGAGAC
Dcg1143 rv	TGAACGCTGAATCATGGC
Construction of pPREx2-*pxpABC*	
pPREx2-cg1141 fw	TGCAGAAGGAGATATACATATGACCACCATCGATCTCAAC
pPREx2-cg1139 rv	AAACGACGGCCAGTGAATTCTTAAAGCAATTTAAATCTGACTTTATC
Construction of pPREx2-*pxpTABC*	
pPREx2-cg1142 fw	TGCAGAAGGAGATATACATATGGCCGACAACAAAAATG
pPREx2-cg1139 rv	AAACGACGGCCAGTGAATTCTTAAAGCAATTTAAATCTGACTTTATC
Construction of pPREx2-*pxpT*	
pPREx2-cg1142 fw	TGCAGAAGGAGATATACATATGGCCGACAACAAAAATG
pPREx2-cg1142 rv	AAACGACGGCCAGTGAATTCTTAACTGCTAAGAAGATCGAAG
Construction of pPREx2-*pxpR*-Strep	
cg1143-Strep fw	TGCAGAAGGAGATATACAtATGACGCAGGCAATAGCAG
cg1143-Strep rv	GAACTGTGGGTGGGACCAGCTAGCAAATTCCGGTAGGTGCGCAG
Construction of pJC1-P*_pxpT_*-venus	
Pcg1142 fw	TCAGCGACGCCGCAGGGGATGTGACTGATCTTAAAGGAC
Pcg1142 rv	CATGATATCCCTCCTCTAAGTGCTGGCTGAGACTAGCTG
RBS-Venus fw	TAGAGGAGGGATATCATGGTG
venus-rv	GTTGCCATTGCTGCAGGTCGAC
Construction of pJC1-P*_pxpT_*-Venus-mut1	
PxpT_mut_F	aggccacccggtggcctGCGATTGGTGTGCAATCTACCCC
PxpT_mut_R	aggccaccgggtggcctTGGTGAGTTGAGAGTGAGTTTAG
Construction of pJC1-P*_pxpT_*-Venus-mut2	
PxpT_del_F	GTCTAAACTCACTCTCAACTCACCAGCGATTGGTGTGCAATCTACCCC
PxpT_del_R	GGGGTAGATTGCACACCAATCGCTGGTGAGTTGAGAGTGAGTTTAGAC

### Growth experiments in BioLector microcultivation systems

Growth experiments with *C. glutamicum* strains were routinely performed as microscale cultivations with BioLector I or II instruments (Beckman Coulter, Brea, USA). Growth in this system was measured online as backscattered light at 620 nm (66). Cultivations were performed in a 48-well FlowerPlate (Beckman Coulter, Brea, USA) by using the defined CGXII media described above. When strains carried expression plasmids, 25 µg/mL kanamycin was added to the medium. Inoculations were individually prepared to reach an initial OD_600_ of 1.0. BioLector cultivations were performed at 30°C, 1,200 rpm, and 85% humidity. The specific fluorescence measurements were taken from endpoint BioLector cultivations measured as fluorescence (Ex_508_/Em_532_)/backscatter on the BioLector system or as fluorescence (Ex515/Em528 − bandwidth 5 nm)/OD_600_ on a Tecan Infinite M1000 Pro microplate reader (Tecan Group Ltd, Männedorf, Switzerland).

### Standard recombinant DNA methods and construction of deletion mutants

Standard methods such as PCR and plasmid restriction were carried out according to established protocols (67). All oligonucleotides used are listed in Table 2. Plasmids were constructed by ligating DNA fragments obtained by restriction digestion or PCR or by Gibson assembly (68). Oligonucleotide synthesis and DNA sequencing were performed by Eurofins Genomics (Ebersberg, Germany). Transformation of *E. coli* was performed using a standard protocol (60), and *C. glutamicum* transformation was performed by electroporation (69). *C. glutamicum* deletion mutants were constructed by double homologous recombination using pK19mobsacB-based plasmids as described previously (70). Oligonucleotides annealing upstream and downstream of the deleted genes were used to confirm genomic deletions by colony PCR.

### PxpR overproduction and purification

For testing the binding of 5-OP and other metabolites to PxpR, the protein was overproduced in *E. coli* BL21(DE3) using the expression plasmid pPREx6-*pxpR*-Strep encoding a PxpR protein with a C-terminal Strep-tag-II. The strain was cultivated in TB medium with 50 μg/mL kanamycin at 37°C. At an OD_600_ of about 0.6, *pxpR* expression was induced by addition of 0.5 mM IPTG, and then the culture was further incubated overnight at 18°C. Afterward, cells were harvested and stored at −80°C. For purification of PxpR, cells were resuspended (4 g cell wet weight/mL) in Strep binding buffer (100 mM Tris-HCl, 150 mM NaCl, and 1 mM EDTA; pH 8) supplemented with cOmplete EDTA-free protease inhibitor cocktail (Roche, Basel, Switzerland) and disrupted by one passage through a Multishot Cell Disruptor (Constant System, Georgia, USA) at 20,000 psi. The cell extract was first subjected to low-speed centrifugation (5,000 × *g*, 4°C, 20 min) and subsequent ultracentrifugation of the supernatant (100,000 × *g*, 4°C, 1 h). Supernatants of the ultracentrifugation were loaded onto a StrepTrap HP column (GE Healthcare, Chicago, IL, USA) and, after washing, the Strep-tagged protein was eluted using binding buffer containing 2.5 mM desthiobiotin. The protein was further purified by size exclusion chromatography on a Superdex 200 10/300 GL column (GE Healthcare, Chicago, IL, USA) equilibrated in HEPES buffer (40 mM HEPES-NaOH and 100 mM NaCl; pH 7.4). Protein concentrations were determined using a Colibri microvolume spectrometer (Berthold Detection Systems GmbH, Pforzheim, Germany), and the molar extinction coefficient at 280 nm of 22,920 M^−1^ cm^−1^ was predicted by the ProtParam tool (http://web.expasy.org/protparam/).

### Isothermal titration calorimetry

Purified PxpR-Strep was dialyzed overnight in HEPES buffer (40 mM HEPES-NaOH, pH 7.4, 100 and mM NaCl). Stock solutions (20 mM) of 5-OP, L-glutamic acid, L-glutamine, L-proline, and L-aspartic acid were prepared in dialysis buffer, and the pH was adjusted to pH 7.4 using NaOH. ITC measurements were performed with a MicroCal PEAQ-ITC instrument operated at 25°C. The protein concentration was 40 μM, and the ligand concentration was 150 μM. Prior to filling the measuring cell with 300 μL protein solution, the cell was rinsed with dialysis buffer, and the syringe was filled with 75 μL ligand solution. An ITC run was started with an initial injection of 0.4 μL followed by 36 injections of 1 μL each. In addition, control experiments with the ligand solution titrated into the dialysis buffer were performed. The data were analyzed using the MicroCal ITC analysis software (Malvern Panalytical, Malvern, United Kingdom).

## Data Availability

Data supporting the study findings are available from the corresponding author upon reasonable request.

## SUPPLEMENTAL MATERIAL

Supplemental material aem.02181-25-s0001.pdf Table S1 and Fig. S1.

ASM does not own the copyrights to Supplemental Material that may be linked to, or accessed through, an article. The authors have granted ASM a non-exclusive, world-wide license to publish the Supplemental Material files. Please contact the corresponding author directly for reuse.
