# Supplementary material for: Growth of Corynebacterium glutamicum on 5-oxo-L-proline (pyroglutamate) as a carbon and nitrogen source requires the PxpR-controlled pxpTABC genes
Source: Appl Environ Microbiol. 2026 May 29;92(6):e02181-25. doi: 10.1128/aem.02181-25 (PMC13274462; doi:10.1128/aem.02181-25)
Supplement: Supplemental material — Table S1 and Fig. S1. [file aem.02181-25-s0001.pdf]

Supplementary information to Sundermeyer et al. (2026)

Table S1. Thermodynamic parameters of 5-OP binding to PxpR-Strep at 25°C in 40 mM HEPES-NaOH buffer pH 7.4 containing 100 mM NaCl.

Experiment number	PxpR (µM)	5-OP (µM)	Binding stoichiometry	K_D (nM)	ΔH (kJ mol^{-1})	ΔG (kJ mol^{-1})	$-T\Delta S$ (kJ mol^{-1})
1	40	150	0.325	723	-97.7	-35.1	62.6
2	40	150	0.322	746	-98.0	-35.0	63.0
3	40	150	0.318	688	-98.1	-35.2	62.8
4	40	150	0.330	742	-99.8	-35.0	64.8
5	40	150	0.325	729	-99.7	-35.1	64.6
Average			0.324 ± 0.004	726 ± 23	-98.7 ± 1.0	-35.1 ± 0.1	63.6 ± 1.1

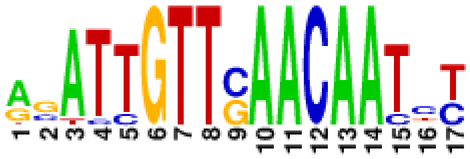

Fig. S1. DNA-binding motif of PxpR proposed by RegPrecise (42).
